# HPV, Cytology, and Cotest Cervical Cancer Screening and the Risk of Precancer

**DOI:** 10.1001/jamanetworkopen.2026.1304

**Published:** 2026-03-11

**Authors:** Anna Gottschlich, Laurie W. Smith, Quan Hong, Smritee Dabee, Lovedeep Gondara, Darrel Cook, Ruth Elwood Martin, Joy Melnikow, Stuart Peacock, Lily Proctor, Gavin Stuart, Eduardo L. Franco, Mel Krajden, Gina S. Ogilvie

**Affiliations:** 1 Women’s Health Research Institute, BC Women’s Hospital and Health Services, Vancouver, British Columbia, Canada; 2 Department of Oncology, Wayne State University, Detroit, Michigan; 3 Population Sciences and Disparities Research Program, Barbara Ann Karmanos Cancer Institute, Detroit, Michigan; 4 Faculty of Medicine, University of British Columbia, Vancouver, British Columbia, Canada; 5 British Columbia Cervix Screening Program, BC Cancer, Vancouver, British Columbia, Canada; 6 British Columbia Centre for Disease Control, Vancouver, British Columbia, Canada; 7 Population Health Sciences, BC Cancer, Vancouver, British Columbia, Canada; 8 Faculty of Health Sciences, Simon Fraser University, Vancouver, British Columbia, Canada; 9 Division of Cancer Epidemiology, McGill University, Montreal, Quebec, Canada; 10 Center for Healthcare Policy and Research, University of California, Davis, Sacramento, California

## Abstract

**Importance:**

There is a global call to end cervical cancer, and various jurisdictions are still determining optimal strategies to accelerate elimination. Human papillomavirus (HPV)-negative testing confers lower risk of future precancer vs normal cytology; high-quality longitudinal data are needed comparing risk after a negative HPV test vs negative cotest (HPV and cytology).

**Objective:**

To compare long-term risk of cervical precancer based on HPV, cytology, and cotest screening results.

**Design, Setting, and Participants:**

This cohort study linked data from a randomized clinical trial to a comprehensive screening program in British Columbia. Participants were recruited between 2006 and 2012 and followed from trial exit to 10 years postexit. Eligible participants were women who completed trial exit cotesting. Data were analyzed between January and April 2025.

**Exposure:**

HPV and cytology status from exit cotesting were considered, stratified by status of each test.

**Main Outcome and Measures:**

Cumulative risk of precancer was calculated over follow-up using Kaplan-Meier techniques. Risk was compared among groups who tested HPV-negative with normal cytology, HPV-negative with abnormal cytology, HPV-positive with normal cytology, and HPV-positive with abnormal cytology. Additionally, risk among those who were HPV-negative (regardless of cytology result), with normal cytology (regardless of HPV results), or were cotest negative were compared in order to simulate outcomes in primary HPV screening, cytology, and cotest programs, respectively.

**Results:**

In this cohort of 8078 women (median [IQR] age at exit screen, 49 [41-57] years; 1636 Asian [22.4%], 223 Indigenous [3.0%], 5568 White [76.1%]) who participated in a British Columbia–based cervical cancer screening trial, the HPV-positive with abnormal cytology group had the highest cumulative incidence risk (CIR) of cervical intraepithelial neoplasia grade 2 or higher at the end of follow-up (CIR, 43.47%; 95% CI, 23.45%-58.26%), followed by the HPV-positive and cytology-negative group (CIR, 22.21%; 95% CI, 11.49%-31.62%). The HPV-negative with abnormal cytology (CIR, 4.83%; 95% CI, 0%-10.03%) and the HPV-negative with normal cytology (CIR, 0.37%; 95% CI, 0.13%-0.60%) groups had significantly lower CIR at the end of follow-up. Less than 1% of the population was HPV-negative with abnormal cytology (69 of 8078 [0.85%]). Women who were HPV-negative regardless of cytology results (CIR, 0.41%; 95% CI, 0.17%-0.65%) had a similar risk as those who cotested negative (CIR, 0.37%; 95% CI, 0.13%-0.60%); both groups had lower risk than those with normal cytology results (regardless of HPV result) (CIR, 1.28%; 95% CI, 0.78%-1.78%) throughout follow-up.

**Conclusions and Relevance:**

In this cohort study of cervical cancer screen testing approaches and risk of cervical precancer, after a negative HPV test (regardless of cytology results) risk of precancer remained acceptably low throughout long-term follow-up. This suggests that cotesting yielded limited benefits, while increasing costs, relative to primary HPV testing.

## Key Points

**Question:**

Is the addition of cytology to primary human papillomavirus (HPV) testing associated with improvement in cervical cancer screening?

**Findings:**

In this cohort study of cervical cancer screen testing approaches and risk of cervical precancer, after a negative HPV test (regardless of cytology results) the risk of precancer remained acceptably low (cumulative incidence risk, 0.41) throughout long-term follow-up.

**Meaning:**

These results suggest that compared with primary HPV testing for cervical cancer screening, cotesting may yield limited benefits while increasing costs.

## Teaser

This cohort study compares rates of long-term cervical precancer based on results of cytology, human papillomavirus (HPV) testing, and cotesting during cervical cancer screening.

## Introduction

Over 300 000 women die worldwide from cervical cancer annually.^1^ However, cervical cancer is preventable, due to effective primary (human papillomavirus [HPV] vaccination)^2^ and secondary (cervix screening)^3^ prevention methods. The World Health Organization has thus called for elimination of the disease as a public health problem by the end of the century.^4^ To achieve this ambitious goal, jurisdictions are determining effective vaccination and screening strategies for their respective populations. There are currently multiple recommended screening strategies available,^5,6^ and detailed comparisons of existing recommendations are needed to assist in determining optimal implementation methods.

Historically, cytology-based screening has been used to screen for cervical cancer, particularly in high-resource settings. However, HPV testing has been convincingly demonstrated to be more sensitive than cytology for detecting cervical precancer.^7,8^ Thus, globally, health systems are transitioning to cervical cancer screening programs that include an HPV testing component.^9,10^ Some screening programs have implemented cotesting^11^ (HPV and cytology). The US Preventive Services Task Force recommends primary HPV screening for women aged 30 to 65 years but recommends both cytology and cotesting as alternatives.^5,12^

Recent evidence has raised questions about the utility of cotesting where primary HPV screening is feasible. Our prior work among cotested women demonstrated that minimal disease was captured by cytology^13^ while undetected by HPV. While there is some convincing evidence to support primary HPV screening vs cotesting, most available robust, longitudinal research on cervical screening methods compares cytology with cotesting^8^ and/or cytology to primary HPV screening,^7^ rather than cotesting with primary HPV screening. Isidean et al^14^ described risk of cervical precancer among a smaller cohort (5754 participants) up to 10 years after a cotest and Dillner et al^15^ described risk in a larger cohort up to 6 years after cotesting. Both studies considered HPV results alone, cytology results alone, or a combination of the 2, finding low risk of cervical precancer over follow-up after a negative HPV test, regardless of cytology results. Cotesting is inherently more costly than primary HPV screening^16^ due to an additional, labor-intensive test. Thus, it is critical for program planners to understand the magnitude of any additional benefits provided by the cytology component of cotesting. In this study, we provided updated and extended results to complement that of previously mentioned studies: we compared 10-year risk of cervical precancer in a cohort of women who received cotesting on exit from HPV For Cervical Cancer Control (HPV FOCAL) randomized clinical trial^17^ (including 8078 participants), stratified by test results (ie, considering HPV test results alone, cytology alone, or both) and age groups.

## Methods

The FOCAL-DECADE cohort study was an extension of HPV FOCAL.^17^ In the following analyses, the main outcome was risk of disease detection over the 10-year follow-up period after the trial exit cotest. We compared risk of disease among participants with HPV-negative results (regardless of cytology results), those with a normal cytology screen (regardless of HPV results), and cotest-negative participants (considering the results of both tests) to assess any added benefits of cotesting compared with primary HPV screening. HPV FOCAL participants provided written informed trial consent as well as consent to (1) be recontacted and (2) have personal data linked to other provincial health databases. Ethics approval was obtained from the University of British Columbia Clinical Research Ethics Board. Results reporting adhered to Strengthening the Reporting of Observational Studies in Epidemiology (STROBE) reporting guidelines.

### Study Design and Data Collection

HPV FOCAL^17^ was a 3-group randomized clinical trial that recruited 25 223 routinely screened women ages 25 to 65 years from the general cervical cancer screen eligible population of metropolitan-area Vancouver and Greater Victoria, British Columbia, Canada between 2008 and 2012. Participants were followed for up to 48 months, with the main outcome being risk of disease detection at exit testing. The intervention group (9552 participants) received HPV screening at baseline and cotesting at trial exit. The control group (9457 participants) received cytology screening at baseline and 24 months (parallel to the provincial screening recommendations at the time of the trial) and cotesting at trial exit. Additionally, a safety group (6214 participants) was included. To ensure a complete census of cases detected at exit in the intervention and control groups, any abnormalities (on either test) were immediately referred to colposcopy.

Upon exit from the trial, participants returned to routine screening within the provincial Cervical Screening Program (CSP). The CSP develops and manages cervical screening guidelines within British Columbia and maintains a centralized database, capturing dates and results from 100% of screening tests, follow-up colposcopy, and treatments conducted in the province.^18^ Data from the trial were linked to the CSP registry to create the FOCAL-DECADE cohort, which has been passively followed for 10 years since the conclusion of the trial. Standard of care for cervical screening in British Columbia was cytology testing, until the CSP transitioned to primary HPV screening in January 2024.

For these analyses, we included only participants from the control group who completed trial exit cotesting (eFigure in Supplement 1). This subpopulation received their first HPV screen at exit cotest.

### Variable Creation and Primary Outcomes

The primary outcome was risk of disease detection over the 10-year follow-up period. Disease was defined as cervical intraepithelial neoplasia (CIN) grade 2 or greater as this is the threshold for treatment adopted by most professional guidelines^19,20^ and screening programs, including the CSP.^18^

Exit cotest results from included participants were summarized as detailed below. HPV tests were categorized as positive or negative for high-risk HPV. Cytology screens were categorized as normal or abnormal (ie, abnormal squamous cells of undetermined significance [ASCUS] or greater). Participants were stratified by their exit cotest results as: (1) cotest negative (ie, cytology screen normal and HPV test negative); (2) cytology screen abnormal and HPV test negative; (3) cytology screen normal and HPV test positive; and (4) cytology screen abnormal and HPV test positive. Additionally, overlapping groups were created to mimic populations considered to have a “normal screen” under different screening scenarios. These groups included: (1) cytology screen normal, regardless of HPV results (to simulate a cytology screening program); (2) HPV test negative, regardless of cytology results (to simulate a primary HPV screening program); and (3) cytology screen normal and HPV test negative (to simulate a cotesting program).

Data on sociodemographic variables were collected from the trial baseline. These variables included age (years), defined as age at exit from trial; self-reported race and/or ethnicity (categorized as Asian [Chinese, Filipino, Japanese, Korean, Southern Asia, Southeast Asia, Western Asia], Indigenous, White [British, Eastern European, French, Northern Europe, Southern Europe, Western Europe], and other [Arab, Black, Latin American, other]); education level (categorized as high school or less, trade school or college, or university); number of lifetime sexual partners (categorized as 0 to 1, 2 to 10, 10 or more); and ever smoker (yes or no). Demographic variables were assessed to ensure the selected population was representative of the province. Additionally, years of follow-up in the FOCAL-DECADE cohort, as well as any positive cytology screen during the trial, were collected from trial records.

### Statistical Analyses

Characteristics of included participants were summarized using medians with IQRs and frequencies with proportions, both overall and stratified by exit cotest results. Differences across categories of cotest results were tested using either a 1-way ANOVA (age at exit screen, ethnicity, years of follow-up), Fisher exact test (CIN grade 2 or greater detection, prior positive screen), or χ^2^ test (education level, number of sexual partners, ever smoker). Follow-up started at date of exit cotest (time zero), and the follow-up period was calculated as the difference between the date follow-up started and the date of CIN grade 2 or greater detection (for failures) or the most recent screening test or follow-up examination (for censors). Cumulative incidence of CIN grade 2 or higher over the follow-up period was plotted using *1 − S(t)* Kaplan-Meier curves for the mutually exclusive cotest results groupings (ie, HPV-negative with normal cytology, HPV-negative with abnormal cytology, HPV-positive with normal cytology, and HPV-positive with abnormal cytology). This was also done for the overlapping screening scenario groups (ie, with normal cytology, HPV negative, cotest negative) by overlaying the independent Kaplan-Meier plots to create the figure. Curves were plotted for the overall population and stratified by age group (28 to 35, 36 to 59, and 60 to 69 years). Age groups were selected because screening recommendations often differ for the youngest and oldest screeners. We described number at risk at time zero and total number of events over follow-up overall and by age group. For mutually exclusive cotest results groups, we calculated and compared cumulative incidence risk (CIR) ratios (RR) and risk differences (RD) based on the Kaplan-Meier estimator, with 95% CIs at time points over the follow-up period generated using the delta method. Finally, we calculated the number needed to harm (NNH), defined as the number of patients needed to treat (in this case with colposcopy and biopsy) among the different abnormal screen combinations to identify one additional CIN grade 2 or higher. Cervical cancer screening has demonstrated comparable performance across populations of varying demographics over the age of 30 years, thus models were not adjusted. Analyses were conducted in R version 2023.06.1 (R Project for Statistical Computing). The threshold used for statistical significance was *P* < .05 in 2-sided tests.

## Results

The HPV FOCAL trial recruited 9457 control group participants. Of those, 8078 completed exit cotesting and were included in these analyses (median [IQR] age, 49 [41-57] years; 1636 Asian [22.4%], 223 Indigenous [3.0%], 5568 White [76.1%]). These included 7565 (93.6%) who were HPV negative with normal cytology results, 69 (0.9%) who were HPV-negative with abnormal cytology, 304 (3.8%) who were HPV-positive with normal cytology, and 140 (1.7%) who were HPV-positive with abnormal cytology (eFigure in Supplement 1). The median (IQR) age at the exit cotest was lower in the HPV-positive with abnormal cytology group than the full cohort (40 [33-48] years vs 49 [41-57] years) (Table 1). Demographic characteristics were similar to the complete trial control group.^17^ For education levels, 1240 participants (17.0%) had a high school education or less, 2132 (29.3%) had trade school or college education, and 3904 (53.7%) attended university (Table 1). A higher percentage in the HPV-positive with abnormal cytology group had a prior positive cytology result in the trial (26 of 140 [18.6%]) and subsequently had CIN grade 2 or higher detection throughout follow-up (44 of 140 [31.4%]).

**Table 1.** Characteristics of Participants of the Control Group of HPV FOCAL Who Completed Exit Screening, Stratified by Exit Screening Result

Characteristic	Participants, No. (%)					*P* value
	Overall (n = 8078)	HPV−/normal cytology (n = 7565)	HPV−/abnormal cytology (n = 69)	HPV+/normal cytology (n = 304)	HPV+/abnormal cytology (n = 140)	
Age at exit screen, median (IQR), y	49 (41-57)	49 (42-57)	46 (41-51)	48 (37-56)	40 (33-48)	<.001^a^
Ethnicity^b^^,^^c^						
Asian	1636 (22.4)	1524 (22.2)	15 (23.8)	64 (24.4)	33 (27.5)	.80^a^
Indigenous	223 (3.0)	206 (3.0)	2 (3.2)	13 (5.0)	2 (1.7)	.78^a^
White	5568 (76.1)	5243 (76.3)	46 (73.0)	192 (73.3)	87 (72.5)	.004^a^
Other	329 (4.5)	308 (4.5)	3 (4.8)	13 (5.0)	5 (4.2)	.94^a^
Missing	763	695	6	42	20	NA
Education level						
High school or less	1240 (17.0)	1164 (17.0)	6 (9.7)	48 (18.0)	22 (18.0)	.18^d^
Trade school or college	2132 (29.3)	1979 (29.0)	22 (35.5)	95 (35.8)	36 (29.5)	
University	3904 (53.7)	3684 (54.0)	34 (54.8)	122 (46.0)	64 (52.5)	
Missing	802	738	7	39	18	
No. of lifetime sexual partners						
0-1	1760 (24.4)	1706 (25.2)	14 (23.7)	25 (9.6)	15 (12.3)	<.001^d^
2-10	3971 (55.1)	3709 (54.8)	34 (57.6)	153 (58.5)	75 (61.5)	
>10	1477 (20.5)	1352 (20.0)	11 (18.6)	82 (31.5)	32 (26.2)	
Missing	870	798	10	44	18	
Ever smoker						
Yes	4641 (64.1)	4370 (64.3)	45 (73.8)	146 (55.7)	80 (66.7)	.01^d^
No	2596 (35.9)	2424 (35.7)	16 (26.2)	116 (44.3)	40 (33.3)	
Missing	841	771	8	42	20	
Positive cytology screen in 4 y prior to exit screen	283 (3.5)	221 (2.9)	9 (13.0)	27 (8.9)	26 (18.6)	<.001^e^
Follow-up time after exit screen, median (IRQ), y	6.6 (5.3-7.8)	6.6 (5.3-7.8)	6.3 (4.3-8.1)	6.6 (4.9-7.7)	5.5 (1.3-7.2)	<.001^a^
Abnormal CIN result grade (grade ≥2)	100 (1.2)	15 (0.2)	3 (4.3)	38 (12.5)	44 (31.4)	<.001^e^
Grade 2	46 (46.0)	6 (40.0)	1 (33.3)	21 (55.3)	18 (40.9)	.99^d^
Grade ≥3	54 (54.0)	9 (60.0)	2 (66.7)	17 (44.7)	26 (59.1)	NR

Abbreviations: CIN, cervical intraepithelial neoplasia; HPV, human papillomavirus.

^a^ One-way ANOVA.

^b^ Participants could select none or multiple ethnicities; total does not add to 100%.

^c^ Groups were categorized as follows: Asian (Chinese, Filipino, Japanese, Korean, Southern Asia, Southeast Asia, Western Asia), Indigenous, White (British, Eastern European, French, Northern Europe, Southern Europe, Western Europe), and other (Arab, Black, Latin American, other).

^d^ χ^2^ test.

^e^ Fisher exact test.

**Figure 1.**
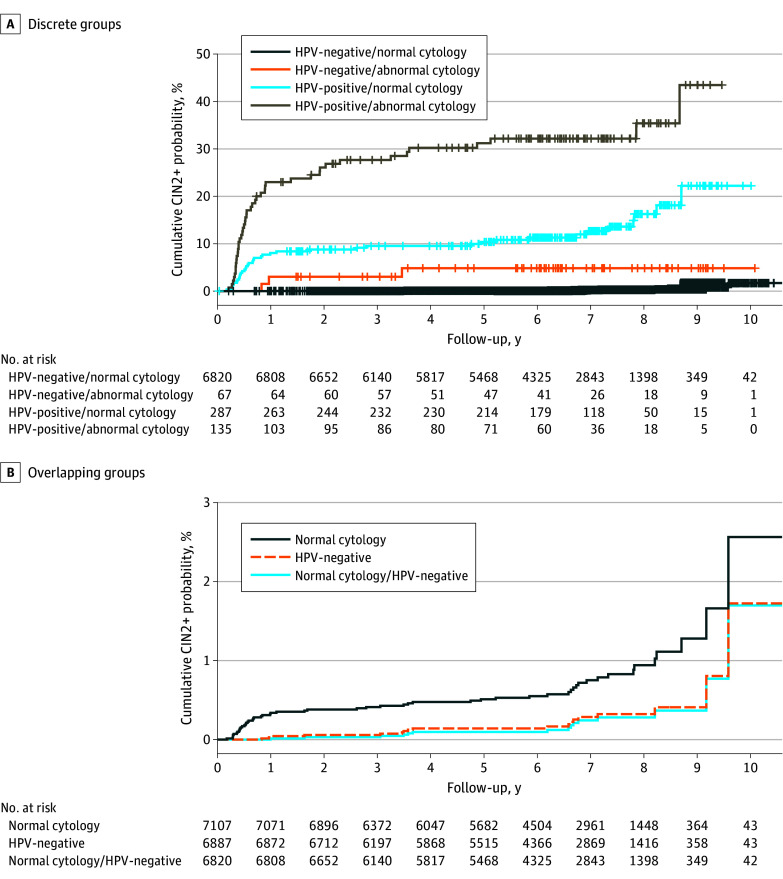
Kaplan-Meier Plots of Cumulative Event Probability for CIN Grades 2 or Higher for Up to 10 Years of Follow-Up by Status Log-rank test *P* value < .001.

Figure 1 shows the cumulative incidence risk for detection of CIN grade 2 or higher over follow-up, stratified by cotest results. The group testing HPV-positive with abnormal cytology had the highest risk of abnormal CIN detection, followed by HPV-positive with normal cytology. The HPV-negative with abnormal cytology and the HPV-negative with normal cytology groups had lower risk. This was similarly demonstrated with the Cox proportional hazard model (eTable 1 in Supplement 1). For overlapping groups, those with normal cytology results (regardless of HPV results) had a higher risk, while the HPV-negative (regardless of cytology results) and cotest-negative groups had similar long-term risk of precancer detection.

**Figure 2.**
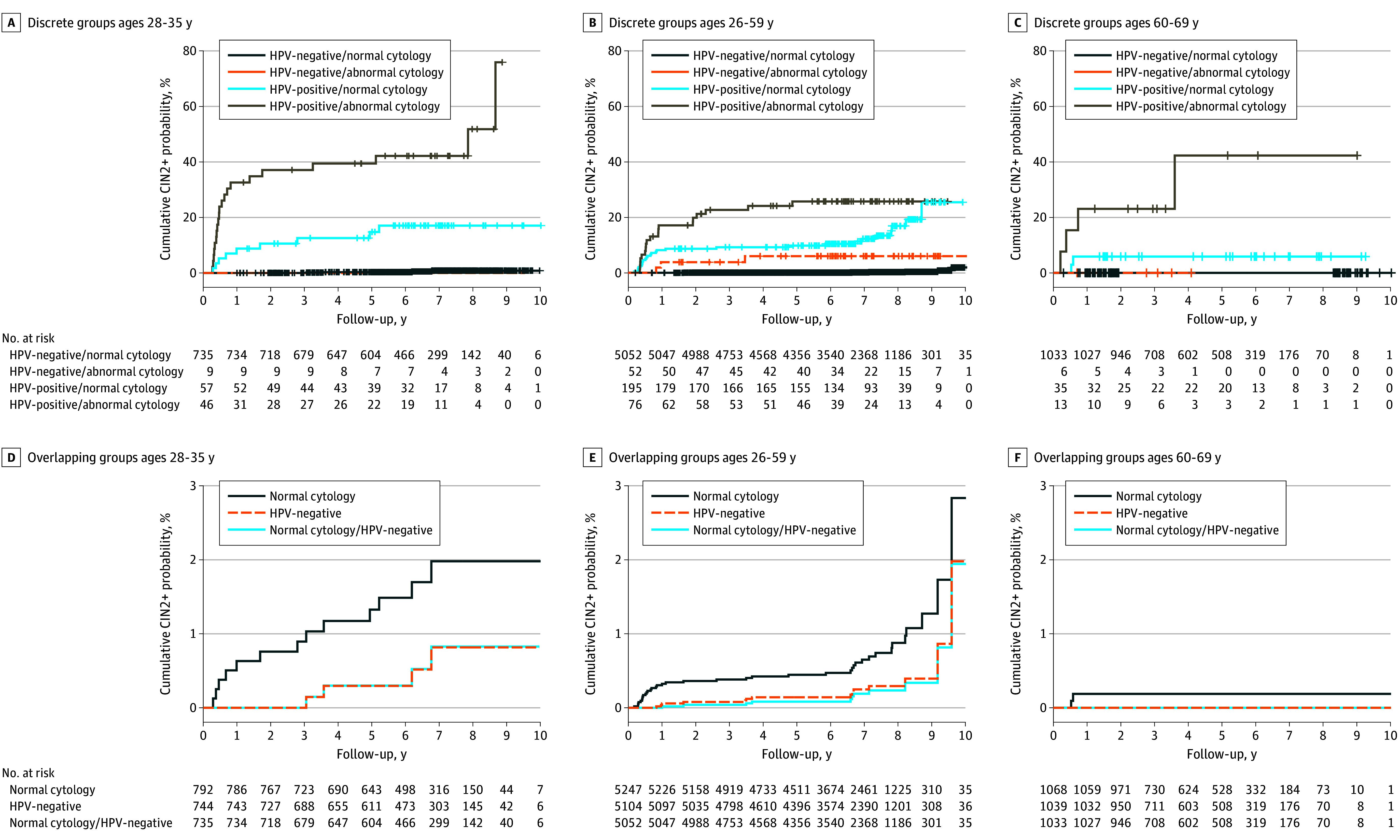
Kaplan-Meier Plots of Cumulative Event Probability for CIN Grades 2 or Higher for Up to 10 Years of Follow-Up by Age Log-rank test *P* values < .001

Similar findings were observed when stratified by age group (Figure 2). Detection of CIN grades 2 and higher occurred earlier in follow-up for the youngest age group compared with the middle group, which was largely driven by a late increase in detection among the HPV-positive with normal cytology group. The cumulative incidence risks in the oldest age group remained significantly lower than the other age groups throughout follow-up. Notably, for the 28-to-35-year-old group, there were no CIN grade 2 or higher detections among HPV-negative with abnormal cytology group (9 individuals) and in the oldest group, there were no detections of CIN grade 2 or higher in either the HPV-negative with abnormal cytology group (6 individuals) or HPV-negative with normal cytology group (1033 individuals) (eTable 2 in Supplement 1).

CIR was calculated at different points over follow-up for each group and RR and RD were summarized for mutually exclusive groups in Table 2. The cumulative incidence risk among HPV-positive with abnormal cytology results was the highest after 9 years of follow-up with a cumulative incidence risk of 43.5% (Table 2). This was followed by HPV-positive with normal cytology results (22.21%; 95% CI, 11.49% to 31.62%), then by HPV-negative with abnormal cytology results (4.83%; 95% CI, 0% to 10.03%), followed by HPV-negative with normal cytology results (0.37%; 95% CI, 0.13% to 0.60%). When comparing overlapping groups, among those who were HPV-negative (regardless of cytology results) the cumulative incidence risk over 9 years of follow up was 0.41% (95% CI, 0.17% to 0.65%). This was very similar to the risk among those who were cotest negative (0.37%; 95% CI, 0.13% to 0.60%) and lower than among those with normal cytology results (regardless of HPV results: CIR, 1.28%; 95% CI, 0.78% to 1.78%). Small numbers after the 9-year mark led to unstable estimates.

**Table 2.** Absolute and Relative Comparisons of Cumulative Incidence Risk for CIN Grade 2 or Higher by Cotest Screening Results at Intervals Over Follow-Up

Groups	Time after cotest (95% CI)											
	3 y			5 y			7 y			9 y		
	CIR, % (95% CI)	RD	RR	CIR, % (95% CI)	RD	RR	CIR, % (95% CI)	RD	RR	CIR, % (95% CI)	RD	RR
Test result-based (independent)												
HPV negative, normal cytology	0.03 (0.00 to 0.07)	[Reference]	1 [Reference]	0.10 (0.02 to 0.17)	[Reference]	1 [Reference]	0.24 (0.09 to 0.39)	[Reference]	1 [Reference]	0.37 (0.13 to 0.60)	[Reference]	1 [Reference]
HPV negative, abnormal cytology	3.03 (0.00 to 7.08)	3.00 (−2.94 to 8.94)	102.87 (14.71 to 719.40)	4.83 (0.00 to 10.03)	4.73 (−4.73 to 14.19)	50.10 (12.75 to 196.75)	4.83 (0.00 to 10.03)	4.58 (−4.89 to 14.05)	19.94 (5.61 to 70.95)	4.83 (<0.01 to 10.03)	4.46 (−5.03 to 13.94)	13.16 (3.64 to 47.59)
HPV positive, normal cytology	9.53 (6.04 to 12.89)	9.50 (−9.18 to 28.18)	323.50 (77.30 to 1353.84)	10.36 (6.71 to 13.87)	10.26 (−10.04 to 30.57)	107.55 (44.95 to 257.37)	12.66 (8.39 to 16.73)	12.42 (−12.40 to 37.24)	52.32 (26.00 to 105.33)	22.21 (11.49 to 31.62)	21.83 (−21.69 to 65.37)	60.03 (27.44 to 133.66)
HPV positive, abnormal cytology	27.65 (19.65 to 34.86)	27.62 (−26.58 to 81.82)	938.78 (228.59 to 3855.42)	31.20 (22.74 to 38.73)	31.10 (−30.05 to 92.25)	323.83 (139.66 to 750.88)	32.17 (23.58 to 39.79)	31.92 (−31.12 to 94.97)	132.93 (68.24 to 258.91)	43.47 (23.45 to 58.26)	43.11 (−42.10 to 128.31)	118.56 (55.44 to 253.56)
Screening program-based (overlapping)												
Cotest negative	0.03 (0.00 to 0.07)	NA	NA	0.10 (0.02 to 0.17)	NA	NA	0.24 (0.09 to 0.39)	NA	NA	0.37 (0.13 to 0.60)	NA	NA
HPV negative	0.06 (0.00 to 0.12)	NA	NA	0.14 (0.05 to 0.23)	NA	NA	0.29 (0.13 to 0.44)	NA	NA	0.41 (0.17 to 0.65)	NA	NA
Normal cytology	0.41 (0.26 to 0.56)	NA	NA	0.51 (0.34 to 0.68)	NA	NA	0.75 (0.52 to 0.98)	NA	NA	1.28 (0.78 to 1.78)	NA	NA

Abbreviations: CIR, cumulative incidence risk; HPV, human papillomavirus; NA, not applicable; RD, risk difference; RR, risk ratios.

Table 3 shows the NNH to identify 1 additional CIN grade 2 or higher. For those negative for HPV but with abnormal cytology, 25 screening tests were needed to detect 1 additional CIN grade 2 or higher compared with less than 7 for those positive for HPV with normal cytology and only 3 for those positive for HPV with abnormal cytology.

**Table 3.** Number Needed to Harm (NNH) to Identify an Additional Cervical Precancer Across Abnormal Results Compared With Cotest Negative Screening Results

Groups	No.	Events	Experimental event rate	Absolute risk increase	NNH
HPV negative, normal cytology	6820	15	[Reference]	[Reference]	[Reference]
HPV negative, abnormal cytology	67	3	0.04	0.04	25.00
HPV positive, normal cytology	287	38	0.13	0.13	7.69
HPV positive, abnormal cytology	135	44	0.33	0.33	3.03

Abbreviation: HPV, human papillomavirus.

## Discussion

In this analysis, we compared longitudinal risk of cervical precancer after various cervical cancer screening results. We found that the risk of cervical precancer detection 9 years after a negative HPV test (regardless of cytology results) was similar to the risk only 3 years after a normal cytology screen (regardless of HPV results). Cytology is often recommended at 3-year intervals, thus we propose that those who were HPV negative had an acceptably low risk of precancer detection over the entire follow-up period, as did those with a negative cotest, who had an even lower risk. Only 3 out of 100 detections of CIN grade 2 or higher were identified among those with a negative HPV test and an abnormal cytology screen, and the HPV negative with abnormal cytology subgroup comprised less than 1% of the study population. The low risk of CIN grade 2 or higher detection after a negative HPV test, regardless of cytology results, was observed for all age groups. In fact, among women aged 60 to 69 years, there were zero detections in the HPV-negative group (1039 participants) over follow-up.

Multiple randomized trials from Europe and North America in the early 2000s compared use of some combination of cytology, primary HPV screening, and cotesting. The ARTISTIC,^21^ Swedescreen,^22^ NTCC Phase I,^23^ and POBASCAM^24^ studies compared cotesting with cytology alone, while the CCaST,^25^ Compass,^26^ NTCC Phase II,^23^ HPV FOCAL,^17^ and FINNISH^27^ studies compared HPV testing with cytology alone. These trials found screening strategies including HPV testing were more effective at identifying cervical precancer compared with cytology-only strategies, generally by noting a higher number of precancers identified in HPV group(s) at the initial screen and a lower percentage at subsequent rounds.^17,22,24^ Additionally, a study from Ronco et al^28^ combined study populations from ARTISTIC, NTCC I, POBASCAM, and Swedescreen trials and followed them for an average of 6.5 years after trial-based screening. They found screening using HPV provided 60% to 70% greater detection of invasive cervical carcinomas compared with cytology. Based on the findings from these studies and others, it is generally accepted that screening methods that involve an HPV test are preferred over cytology alone.

However, even with the exclusion of cytology-based screening as an optimal method, there is still the choice between primary HPV screening and cotesting, and some major countries, including the US, continue to recommend and employ cotesting. Cotesting involves 2 primary tests, which is more costly to health care systems and detects minimal additional precancers. Given HPV testing has superior sensitivity, it alone detects most cervical precancer.^13^ Primary HPV screening involves 1 test that is less labor-intensive and that can be clinician or self-collected, which enhances testing reach and access and improves screening equity. In 2008, Dillner et al^15^ pooled data from 7 European HPV screening studies and compared the risk of CIN grades 3 or higher over 6 years of follow-up. Their results were similar to ours, there was a very low risk of precancer detection after either a negative cotest or primary HPV screen. They found CIN grade 2 or higher cumulative incidence risk of 0.67% for those negative for HPV and 1.76% for those with normal cytology after 6 years of follow-up. We found CIN grade 2 or higher cumulative incidence risk of 0.41% and 1.30% for those with negative for HPV or with normal cytology, respectively, after 9 years of follow-up, suggesting that risk of precancer after a negative HPV test may remain low after a longer follow-up period.

There were very few women who screened as HPV-negative with abnormal cytology, and this group had only 3 of the total 100 CIN grade 2 or higher detections throughout follow-up. Furthermore, we found that over 3 times the number of diagnostic follow-up tests were needed to detect 1 additional precancer among those who are HPV-negative with abnormal cytology results compared with those who are HPV-positive, regardless of cytology results. In the clinical setting of a population-based, organized cervical screening program, the increased detection from adding cytology to HPV screening would result in substantially increased costs, with minimal improvements in disease detection.

Cotesting not only increases costs with minimal added disease detection, but it also carries potential risks compared with primary HPV testing. Cytology results may be delayed due to the time it takes to process a cytology sample and the human labor involved. In addition, there is a shortage of cytotechnicians in the workforce,^29^ due to decreased demand for cytologists, which slows overall screening. Moreover, women who are HPV positive with abnormal cytology, vs those who are HPV positive with normal cytology, may be prioritized for diagnostics (colposcopy), even though this study found that HPV-positive women face similar risks regardless of their cytology results. Adding cotesting also complicates screening recommendations, which may reduce understanding of and adherence to appropriate follow-up.

This longitudinal study contributes some of the most robust and comprehensive data available to the existing literature comparing the utility of cotesting vs primary HPV screening for cervical cancer. It includes over a decade of follow up data from a large, randomized trial that was linked to an extensive provincial screening registry. The dataset includes data on 100% of screening tests and screening-related tests received in the provincial organized screening program over the entire follow-up, thus minimizing issues with bias and/or confounding due to loss to follow-up.

### Limitations

There are limitations to consider when interpreting the results of this analysis. While data are derived from a randomized trial, this study is a cohort study, as it only includes those from the control group of the trial who completed the trial-exit cotest. Earlier dropouts from the study were not included and comparison groups were created from the control group subpopulation. Additionally, in British Columbia, women currently exit from the screening program at age 69 years. Thus, women who were 60 years or older at exit from the trial may have exited the screening program with a limited amount of follow-up data. This was recorded as a censored observation in our analyses, and we have no way of knowing if they would have had a precancer detected if they had continued screening. However, age restrictions are common in screening programs and demonstrate what the risk of detection looks like in real-world scenarios. Finally, this study was conducted among a screening population, and findings are not generalizable to diagnostic testing among symptomatic women. In the symptomatic population, cotesting may have increased benefits, but that lies outside of the scope of this work and additional studies on this topic are warranted.

## Conclusions

In this longitudinal cohort study of cervical cancer screening approaches and risk of future cervical precancer, a negative HPV result, regardless of cytology status, was associated with a long-term acceptably low risk of cervical precancer detection. Among HPV negative women aged 60 to 69 years, no precancers were detected throughout follow-up, which provides preliminary evidence to suggest that changes to screening recommendations may be appropriate for this group. Findings from this study suggest that the addition of a cytology screen to a standalone HPV screening program (cotesting) provided limited incremental benefits.

## Notes

Supplement 1. eFigure 1. Flow Chart of Subpopulation Selection eTable 1. Hazard Ratios for Test Result-Based Groupings eTable 2. People at Risk and Number of Events Over 10-Year Follow-up by Age Group (Years) and Exit Screen Results

Supplement 2. Data Sharing Statement
