# Supplementary material for: HPV, Cytology, and Cotest Cervical Cancer Screening and the Risk of Precancer
Source: JAMA Netw Open. 2026 Mar 11;9(3):e261304. doi: 10.1001/jamanetworkopen.2026.1304 (PMC12980249; doi:10.1001/jamanetworkopen.2026.1304)
Supplement: Supplement 1. — eFigure 1. Flow Chart of Subpopulation Selection eTable 1. Hazard Ratios for Test Result-Based Groupings eTable 2. People at Risk and Number of Events Over 10-Year Follow-up by Age Group (Years) and Exit Screen Results [file jamanetwopen-e261304-s001.pdf]

Supplemental Online Content

Gottschlich A, Smith LW, Hong Q, et al. HPV, cytology, and cotest cervical cancer screening and the risk of precancer. *JAMA Netw Open.* 2026;9(3):e261304. doi:10.1001/jamanetworkopen.2026.1304

eFigure. Flow Chart of Subpopulation Selection

eTable 1. Hazard Ratios for Test Result-Based Groupings

eTable 2. People at Risk and Number of Events Over 10-Year Follow-up by Age Group (Years) and Exit Screen Results

This supplemental material has been provided by the authors to give readers additional information about their work.

© 2026 Gottschlich A et al. *JAMA Network Open*.

eFigure. *Flow chart of subpopulation selection.* Participants from the Control Arm of the HPV FOCAL trial who completed exit co-testing were included in this analysis (N=8,078). A detailed description of the excluded Control Arm participants as well as the exit co-test results from the included participants is included in the figure.

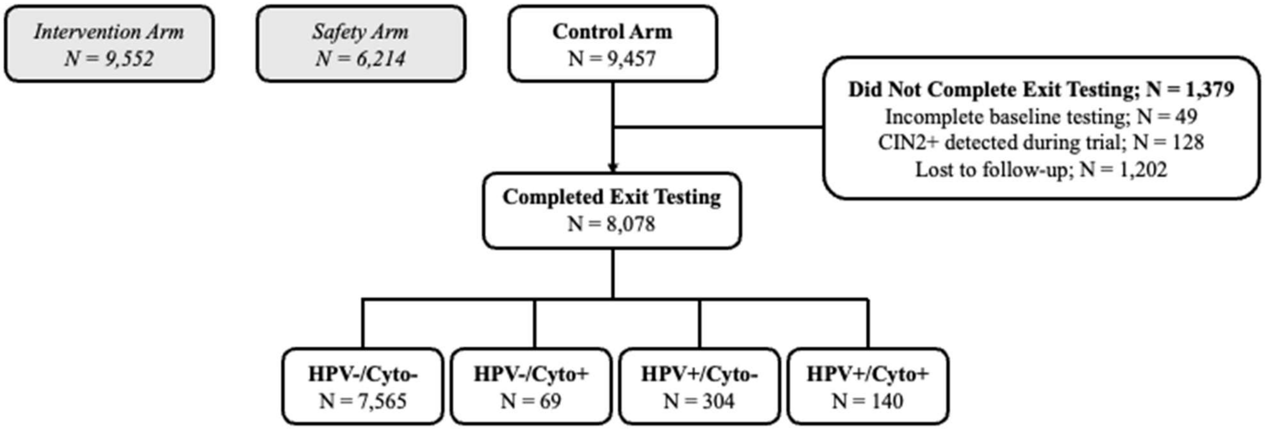

© 2026 Gottschlich A et al. *JAMA Network Open*.

eTable 1. Cox proportional hazard ratios for test result-based groupings

	**Overall**		**28-35 years at exit screen**		**36-59 years at exit screen**		**60-69 years at exit screen**	
	**HR[A]**	**95% CI**	**HR**	**95% CI**	**HR**	**95% CI**	**HR**	**95% CI**
**HPV-/Cyto-**	*Ref*	*Ref*	*Ref*	*Ref*	*Ref*	*Ref*	N/A	N/A
**HPV-/Cyto+**	20.32	(5.9, 70.2)	N/A[B]	N/A	26.56	(7.4, 95.2)	N/A	N/A
**HPV+/Cyto-**	64.75	(35.6, 117.8)	32.51	(10.0, 105.6)	69.72	(34.5, 140.9)	*Ref*	*Ref*
**HPV+/Cyto+**	193.72	(107.5, 349.2)	116.84	(40.0, 341.0)	146.47	(69.3, 309.6)	6.48	(1.17, 35.83)

[A]HR = Hazard ratio

[B]No events occurred in this group.

© 2026 Gottschlich A et al. *JAMA Network Open*.

eTable 2. People at risk and number of events over 10-year follow-up by age group (years) and exit screen results

	Overall (N = 7309)		Age 28-35 (N = 847)		Age 36-59 (N = 5375)		Age 60-69 (N = 1087)	
	At risk at t0	Total events	At risk at t0	Total events	At risk at t0	Total events	At risk at t0	Total events
HPV-/Cyto-	6820 (93.3%)	15 (0.2%)	735 (86.8%)	4 (0.5%)	5052 (94.0%)	11 (0.2%)	1033 (95.0%)	0 (0.0%)
HPV-/Cyto+	67 (1.0%)	3 (4.5%)	9 (1.1%)	0 (0.0%)	52 (1.0%)	3 (6.8%)	6 (0.5%)	0 (0.0%)
HPV+/Cyto-	287 (3.9%)	38 (13.2%)	57 (6.7%)	9 (15.8%)	195 (3.6%)	27 (13.8%)	35 (3.2%)	2 (5.7%)
HPV+/Cyto+	135 (1.8%)	44 (32.6%)	46 (5.4%)	21 (45.7%)	76 (1.4%)	19 (25.0%)	13 (1.2%)	4 (30.8%)

© 2026 Gottschlich A et al. *JAMA Network Open*.
