# Supplementary material for: HPV, Cytology, and Cotest Cervical Cancer Screening and the Risk of Precancer
Source: JAMA Netw Open. 2026 Mar 11;9(3):e261304. doi: 10.1001/jamanetworkopen.2026.1304 (PMC12980249; doi:10.1001/jamanetworkopen.2026.1304)
Supplement: Supplement 2. — Data Sharing Statement [file jamanetwopen-e261304-s002.pdf]

Data Sharing Statement

Gottschlich. HPV, Cytology, and Cotest Cervical Cancer Screening and the Risk of Precancer in the FOCAL-DECADE Cohort. *JAMA Netw Open*. Published March 11, 2026. doi:10.1001/jamanetworkopen.2026.1304

Data
Data available: Yes
Data types: Deidentified participant data
How to access data: The data generated in this study are available upon reasonable request from the corresponding author.
When available: With publication

Supporting Documents
Document types: None

Additional Information
Who can access the data: Data will be made to anyone with a valid scientific proposal after internal review.
Types of analyses: Data will be made to anyone with a valid scientific proposal after internal review.
Mechanisms of data availability: Data will be shared through signed data access agreement with secure transfer protocols.
